# Prevalence and Impact of Malnutrition in Hospitalizations Among Celiac Diseases: A Nationwide Analysis

**DOI:** 10.7759/cureus.44247

**Published:** 2023-08-28

**Authors:** Kanwal Bains, Shivam Kalra, Ishandeep Singh, Jay Patel, Isha Kohli, Mukul Dhiman, Dino Dukovic, Aalam Sohal, Avin Aggarwal

**Affiliations:** 1 Internal Medicine, University of Arizona College of Medicine, Tucson, USA; 2 Internal Medicine, Trident Medical Center, North Charleston, USA; 3 Internal Medicine, Dayanand Medical College and Hospital, Ludhiana, IND; 4 Internal Medicine, Orange Park Medical Center, Orange Park, USA; 5 Public Health Sciences, Icahn School of Medicine at Mount Sinai, New York City, USA; 6 Internal Medicine, Punjab Institute of Medical Sciences, Jalandhar, IND; 7 Internal Medicine, Ross University School of Medicine, Bridgetown, BRB; 8 Hepatology, Liver Institute Northwest, Seattle, USA; 9 Gastroenterology and Hepatology, University of Arizona, Tucson, USA

**Keywords:** national inpatient sample (nis), iron deficiency anemia, gluten sensitivity, celiac disease, s:malnutrition

## Abstract

Background/Aims: Celiac disease (CD) is a T-cell-mediated gluten sensitivity that results in villous atrophy in the small intestine, leading to chronic malabsorption. Patients with celiac disease are prone to malnutrition. We assessed the impact of malnutrition on in-hospital outcomes in patients with CD.

Materials and methods: Patients with a primary discharge diagnosis of CD between January 2016 and December 2019 were included in the National Inpatient Sample Database. Data were collected on patient demographics, hospital characteristics, the Charlson Comorbidity Index (CCI), and concomitant comorbidities. The association between malnutrition and outcomes, including mortality, deep vein thrombosis (DVT), pulmonary embolism (PE), sepsis, acute kidney injury (AKI), length of stay (LOS), and total hospitalization charges (THC), was analyzed using the multivariate regression model.

Results: A total of 187310 patients with CD were included in the analysis. Patients with CD and malnutrition had a higher risk of mortality (adjusted odds ratio [aOR], 2.08; p<0.001), AKI (aOR=1.18, p=0.003), and DVT (aOR=1.53; p<0.001) compared to patients with CD without malnutrition. No significant difference was noted in the rates of sepsis and PE. Patients with malnutrition also had a prolonged LOS (2.89 days; p<0.001) and higher THC ($22252.18; p<0.001) compared to patients without malnutrition.

Discussion: Patients with CD and malnutrition are at high risk of worse outcomes. Early identification of malnutrition in CD can help prevent morbidity and mortality. Even strict adherence to a gluten-free diet has been associated with malnutrition. Further studies identifying factors associated with malnutrition in CD and the impact of interventions to prevent and treat malnutrition are encouraged.

## Introduction

Malnutrition is one of the most common health problems seen in hospitalized patients [1]. It refers to a state of negative energy balance classified by the presence of low BMI, unintentional weight loss, and compromised nutrition intake for >5 days as per certain screening tools like the Malnutrition Universal Screening Tool in hospitalized patients [2]. In 2009, ASPEN (American Society for Parenteral and Enteral Nutrition) and ESPEN (European Society for Clinical Nutrition and Metabolism) provided an etiologic approach to diagnosing adult malnutrition, namely malnutrition secondary to acute disease or injury, chronic disease or injury, and malnutrition related to starvation [3]. This framework emphasizes how inflammation contributes to cytokine-mediated catabolism. The presence of a systemic inflammatory response to an insult or injury contributes to the degree of malnutrition. Consequently, even though nutrition supplementation is an important intervention, muscle loss still occurs under conditions having high levels of inflammation [4].

Semistarvation is another mechanism of malnutrition that is associated with an array of gastrointestinal diseases. This condition results from either inadequate intake or poor assimilation as a consequence of a decrease in the functional intestinal surface [4]. Celiac disease is a chronic inflammatory enteropathy that is more common in those with HLA DQ2 and HLA DQ8 genetic predispositions [5]. It affects people of every age and gender globally, with an estimated prevalence range of 0.7% to 1.4% [6]. Gluten, a protein found in wheat, rye, and barley, causes an adaptive T cell-mediated response that results in villous atrophy, leading to a loss of effective absorption of nutrients and vitamins [7]. As per Food and Drug Administration (FDA) regulation in 2013, foods labeled gluten-free must contain less than 20 parts per million [8].

Clinical manifestations of celiac disease might range from being asymptomatic to malabsorption, causing diarrhea, steatorrhea, weight loss, or edema due to hypoalbuminemia [9]. The most common deficiencies seen in CD patients at diagnosis are iron, vitamin D, calcium, vitamin B12, folic acid, and zinc [7]. Studies examining the impact of malnutrition on hospitalized patients with CD are rare [10]. We hypothesized that malnutrition would be associated with worse outcomes in patients with celiac disease.

## Materials and methods

Data source 

The largest database of inpatient hospital stays in the United States is the National Inpatient Sample (NIS), which is maintained by the Healthcare Cost and Utilization Project (HCUP) [11]. It contains information on 35 million weighted hospitalizations annually. Information regarding this data source has been discussed in previous studies [12,13]. NIS is a de-identified database of every hospitalization, which is maintained as a unique entry with one primary discharge diagnosis and up to 39 additional diagnoses during that hospitalization. Each hospitalization entry contains information about the patient's age, sex, race, insurance status, primary and secondary procedures (up to 25), hospitalization outcome, overall costs, and length of stay (LOS). No IRB approval was required for this study as it was conducted on publicly available, de-identified data. 

Study population

We identified adult patients hospitalized between 2016 and 2019 who had celiac disease as their primary diagnosis using the International Classification of Diseases 10th Version, Clinical Modification (ICD-10 CM) diagnosis codes. The patients were stratified into two groups based on the presence of malnutrition using ICD-10 codes. There were 209,520 cases that were identified. We then excluded patients aged<18 years (n-19,895) and cases with missing data on in-hospital mortality or demographic information (n-6,325). In total, 187,310 cases met the inclusion criteria. 

Study outcomes and variables

The primary outcome of the study was the rates of inpatient mortality between malnourished and non-malnourished patients with CD. Secondary outcomes included rates of deep vein thrombosis (DVT), acute kidney injury (AKI), sepsis, and pulmonary embolism (PE). We also compared the mean LOS and total hospitalization charges between the two groups. The nutritional status of the patient was the primary exposure variable, using ICD-10 codes for malnutrition (E40.x-E46.x, R63.4, R64). These codes have been used to identify malnutrition and failure to thrive in various studies [14-16]. The data was gathered on age groups (split into three categories: <44 years, 45-64 years, and >65 years), gender, race, insurance status, median household income, and hospital features (including region, bed size, and location being either urban or rural). The Charlson Comorbidity Index (CCI) was employed to determine how burdensome comorbidities are in the two study groups [17]. This is an ICD-10-CM-based indicator that has undergone significant validation and is intended to be used in large administrative datasets to forecast mortality and hospital resource consumption.

Statistical analysis

National estimates were generated using hospital discharge weights provided by NIS. Chi-square and independent t-tests were used to compare categorical and continuous variables, respectively. Univariate logistic regression was performed to identify the association between malnutrition and categorical/continuous outcomes. For the variables that met the cut-off of p<0.1 on univariate analysis, multivariate logistic regression was performed while accounting for patient demographics, hospital features, and Charlson comorbidities. The unadjusted and adjusted odds ratios were reported with a 95% confidence interval. A p-value<0.05 was considered statistically significant. STATA 17.0 (Texas) was used for data analysis.

## Results

Patient demographics

A total of 187,310 patients were admitted with CD between 2016 and 2020. Of these, 15,555 (8.3%) patients had a concomitant diagnosis of malnutrition using ICD-10 codes. Most of the patients in the malnutrition group were elderly > 65 years old (53.1%), female (66.3%), had Medicare (60.14%), and were in the highest income quartile (27.9%). These results are presented in Table 1. 

**Table 1:** Patient characteristics, stratified by the presence of malnutrition 'n' indicates the sample size. Values in parentheses denote percentages. 'p-value' indicates the level of statistical significance between groups. Results with a p-value less than 0.05 are considered statistically significant

Demographics	Absence of malnutrition n(%)	Presence of malnutrition n (%)	p-value
Age category			<0.001
18-44	54,775 (31.9)	3,050 (19.6)	
45-64	49,660 (28.9)	4,240 (27.3)	
>65	67,320 (39.2)	8,265 (53.1)	
Sex			<0.001
Males	47,190 (27.5)	5,245 (33.7)	
Females	12,456,5 (72.5)	10,310 (66.3)	
Race			0.1545
White	153,450 (89.3)	13,800 (88.7)	
Black	5,440 (3.2)	525 (3.4)	
Hispanic	8,135 (4.7)	770 (5)	
Asian/Pacific Islander	1,080 (0.6)	160 (1)	
Native American	560 (0.3)	55 (0.4)	
Other	3,090 (1.8)	245 (1.6)	
Primary expected payer			<0.001
Medicare	77,875 (45.34)	9,355 (60.14)	
Medicaid	19,775 (11.5)	1,700 (10.9)	
Private	65,995 (38.42)	3,925 (25.23)	
Uninsured	3,590 (0.1)	295 (1.9)	
Median household income			0.1463
Lowest quartile	31,125 (18.1)	2,890 (18.6)	
Second quartile	41,650 (24.25)	4,015 (25.81)	
Third quartile	48,365 (28.16)	3,405 (27.7)	
Highest quartile	50,615 (29.5)	4,345 (27.9)	
Region of hospital			0.1833
Northeast	44,790 (26.1)	3,865 (24.9)	
Midwest	43,350 (25.2)	4,190 (26.9)	
South	48,085 (28)	4,395 (28.2)	
West	35,530 (20.7)	3,105 (20)	
Hospital Location			0.221
Rural	12,265 (7.1)	1,015 (6.5)	
Urban	159,490 (92.9)	14,540 (93.5)	
Teaching status of the hospitals			0.15
Non-teaching Hospitals	45,550 (26.5)	3,930 (25.2)	
Teaching Hospitals	126,205 (73.5)	11,625 (74.7)	
Bed size of hospital			0.003
Small	35,815 (20.9)	2,830 (18.2)	
Medium	47,880 (27.9)	4,330 (27.8)	
Large	88,060 (51.3)	8,395 (54)	
Charlson Comorbidities			<0.001
0	17,625 (10.3)	0 (0)	
1	26,510 (15.4)	375 (2.4)	
2	30,270 (17.6)	1,255 (8.1)	
>3	97,350 (56.7)	13,925 (89.5)	

Comorbidities

Patients in the malnutrition group had a higher prevalence of many comorbidities such as congestive heart failure, cardiac arrhythmia, valvular disease, pulmonary circulation disorders, peripheral vascular disease, other neurological disorders, chronic pulmonary disease, renal failure, liver disease, peptic ulcer disease, AIDS/HIV, lymphoma, metastatic cancer, solid tumor (without metastasis), coagulopathy, fluid and electrolyte disorder, blood loss anemia, deficiency anemia, alcohol abuse, hypertension (complicated), and smoking compared to patients who did not show malnutrition. A complete list of associated comorbidities in patients who showed malnutrition and those who did not show malnutrition is presented in Table 2. 

**Table 2:** Comorbidities, stratified by the presence of malnutrition 'n' indicates the sample size. Values in parentheses denote percentages. 'p-value' indicates the level of statistical significance between groups. Results with a p-value less than 0.05 are considered statistically significant

Comorbidities	Absence of malnutrition n (%)	Presence of malnutrition n (%)	p-value
Congestive heart failure	22,345 (13)	2,610 (16.8)	<0.001
Cardiac arrhythmia	35,130 (20.4)	4,115 (26.4)	<0.001
Valvular disease	12,840 (7.5)	1,535 (10)	<0.001
Pulmonary circulation disorders	7,280 (4.2)	940 (6)	<0.001
Peripheral vascular disease	10,905 (6.3)	1,460 (9.3)	<0.001
Hypertension, uncomplicated	53,245 (31)	4,375 (28)	<0.001
Paralysis	2,765 (1.6)	195 (1.2)	0.134
Other neurological disorders	18,315 (10.6)	2,470 (16)	<0.001
Chronic pulmonary disease	42,625 (24.8)	4,235 (27.2)	0.004
Diabetes, uncomplicated	18,900 (11)	1,130 (7.2)	<0.001
Diabetes, complicated	23,635 (13.7)	1,990 (12.8)	0.142
Hypothyroidism	41,165 (24)	3,795 (24.4)	0.599
Renal failure	22,280 (13)	2,805 (18)	<0.001
Liver disease	13,040 (7.5)	2,130 (13.7)	<0.001
Peptic ulcer disease, excluding bleeding	2,480 (1.4)	540 (3.4)	<0.001
AIDS/HIV	165 (0.1)	65 (0.4)	<0.001
Lymphoma	2,030 (1.1)	420 (2.7)	<0.001
Metastatic cancer	3,475 (2)	920 (6)	<0.001
Solid tumor (without metastasis)	7,615 (4.4)	1,625 (10.4)	<0.001
Rheumatoid arthritis/collagen vascular disease	13,135 (7.6)	1,305 (8.3)	0.145
Coagulopathy	12,200 (7.1)	1,835 (11.8)	<0.001
Obesity	24,330 (14.1)	610 (3.9)	<0.001
Fluid and electrolyte disorder	52,605 (30.6)	9,385 (60.3)	<0.001
Blood loss anemia	2,140 (1.2)	370 (2.3)	<0.001
Deficiency anemia	13,145 (7.6)	2,460 (15.8)	<0.001
Alcohol abuse	7,975 (4.6)	1000 (6.4)	<0.001
Drug abuse	9,515 (5.5)	890 (5.7)	0.679
Psychosis	2,855 (1.6)	270 (1.7)	0.758
Depression	37,485 (21.8)	3,430 (22)	0.777
Hypertension, complicated	27,780 (16.1)	3,085 (19.8)	<0.001
Smoking	54,550 (31.7)	5,250 (33.7)	0.026

Outcomes

**Figure 1:**
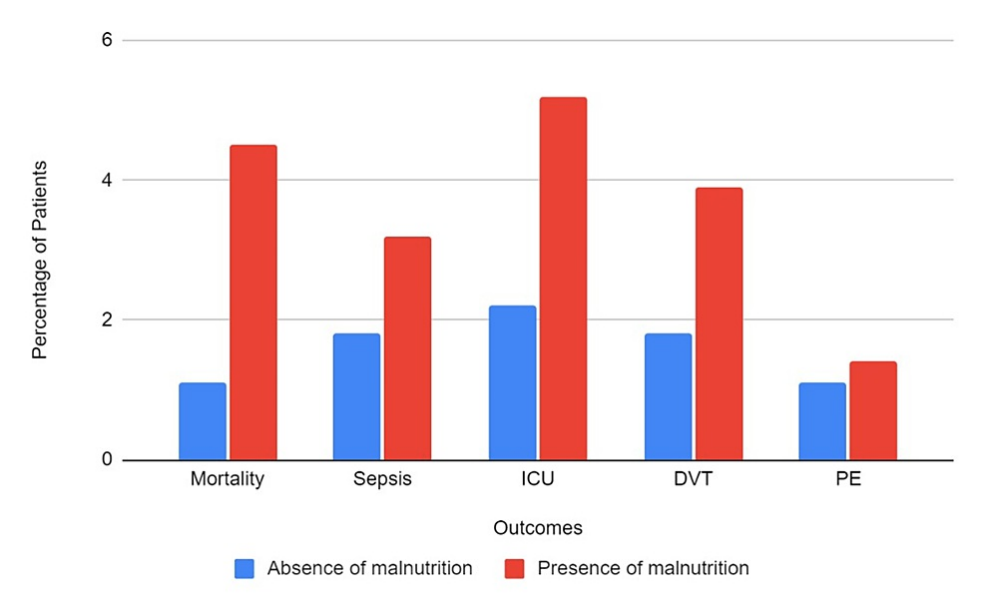
Outcomes in patients with CD, stratified by the presence of malnutrition

In the study population, there were 2,640 (1.41%) total hospital deaths. Patients with malnutrition had a mortality rate of 4.47% as opposed to patients without malnutrition, with a mortality rate of 1.13%. Patients who were malnourished had a statistically significant increased risk of total all-cause mortality (aOR=2.08, 95% CI: 1.68-2.59, p<0.001). The results are displayed in Figure 1. 

The overall incidence of sepsis was about 1.97% of the study population. Sepsis was diagnosed in about 3.21% of patients with malnutrition and 1.86% without malnutrition. Interestingly, there was no statistically significant difference between the two groups on multivariate analysis, with an aOR of 1.13 (95% CI: 0.90-1.43, p-0.3). The incidence of AKI was higher in the malnourished group. It occurred in 24,175 (12.91%) patients overall, and 3,390 (21.79%) patients with malnutrition experienced AKI, as opposed to 20,785 (12.1%) patients who were not malnourished. Malnourished individuals had a statistically significant increased incidence of AKI on multivariate analysis (aOR=1.18, 95% CI- 1.05-1.32, p=0.003). Regarding ICU admission: 4,680 patients, or 2.5%, needed to be admitted to the ICU. Out of these, 805 (5.18%) were malnourished, and 3,875 (2.26%) were from the group without malnourishment. Patients with malnutrition showed a statistically significant higher risk of ICU admission on multivariate analysis (aOR=1.39, 95% CI-1.15-1.68, p=0.001).

DVT occurred in 1.97% of all patients. Patients with malnutrition had a DVT rate of 3.89% as opposed to patients without malnutrition, with a DVT incidence of 1.79%. Patients with malnutrition demonstrated a statistically significant increased risk of DVT on multivariate analysis (aOR=1.53, 95% CI-1.22-1.91, p<0.001). For PE, the overall incidence was 1.17%. In patients with malnutrition, the incidence of PE was 1.41%, compared to 1.15% in patients without malnutrition. In multivariate analysis, the findings between the two groups were not statistically significant (aOR=0.88, 95% CI-0.56-1.36, p-0.6).

Finally, patients with malnutrition, compared to patients without malnutrition, had a higher mean length of stay of 7.93 days (+/-0.17) as opposed to 4.27 days (+/-0.03). Patients with malnutrition had a statistically longer length of stay than those without malnutrition on multivariate analysis (adjusted coefficient=2.89, 95% CI: 2.56-3.22, p<0.001). Similarly, hospitalization costs were higher for malnourished patients, averaging $79,374.61 (+/-2314.12) as opposed to $49,628.86 (+/-482.66) for the group without malnourishment. Again, this difference was statistically significant with an adjusted coefficient of 22252.18 (95% CI: 17,803-26,701, p<0.001).

## Discussion

The prevalence of malnutrition in patients with CD was 8.3% in our study. Similar rates have been seen in other studies [18]. Our study reports that the presence of malnutrition in patients with CD is associated with worse outcomes, such as in-hospital mortality and resource utilization. In general, previous studies have suggested that the presence of malnutrition in hospitalized patients is associated with worse outcomes, including an increased length of stay and an increased risk of mortality [10,19]. Our study results of a 108% higher risk of mortality in patients with malnutrition and CD are in agreement with the prior studies.

The pathophysiology of malnutrition in CD is multifactorial and includes malabsorption secondary to intestinal damage and chronic diarrhea secondary to malabsorption of nutrients, including carbohydrates, proteins, fat, and minerals [20]. Symptoms of malabsorption in patients with CD reflect the tip of the iceberg, while most CD patients do have malnutrition-related symptoms [21]. The most common nutrient deficiencies seen in a CD are iron, zinc, folic acid, vitamin B12, vitamin D, and calcium. A 2012 meta-analysis by M. Tio et al., comprising 38,039 CD patients, shows that CD patients are at an increased risk of all-cause mortality with a pooled OR of 1.24 (95% CI 1.19-1.30) [18]. However, the study suggested that most of the mortality risk in patients with celiac disease was due to increased cardiovascular mortality and the risk of non-Hodgkin lymphoma [22]. The presence of various micronutrient deficiencies has also been linked to mortality in hospitalized patients and patients undergoing major surgeries [23]. Malnutrition may also increase mortality through other mechanisms, such as hypoglycemia and hypothermia [23,24]. Patients with malnutrition who are unable to tolerate oral feeding should receive an alternative form of enteral nutrition to improve outcomes.

Patients with malnutrition were also noted to be at increased risk of DVT. Malnutrition has been reported to be a known risk factor for thrombosis in patients with gastrointestinal conditions [25,26]. Previous studies have shown a significant relationship between the risk of DVT and BMI, weight, and waist circumference. It has also been suggested that diet influences factors VII c, VIII c, and von-Willebrand, which are further related to the risk of thrombosis. Folsom et al. reported that there is a relationship between venous thromboembolism and serum albumin levels. Several small, cross-sectional, or retrospective clinical studies have reported conflicting results [27,28]. It has been reported that low serum albumin can increase fibrinogen and factor VIII levels, resulting in a hypercoagulable state. A deficiency of vitamin D in malnourished patients can also increase the risk of DVT [29]. Furthermore, vitamin B12 and folate deficiency lead to increased production of homocysteine, which has been involved in the thrombotic process [30,31]. Further studies investigating this association are necessary.

Our study is in agreement with previous studies, which have reported that malnutrition is associated with an increased risk of developing AKI [32]. These patients are malnourished and likely dehydrated, and thus, it is possible that the chances of developing AKI are higher than in the healthy population. We also believe that since an elevation of serum creatinine is necessary for the diagnosis of AKI, and in these patients, the levels of serum creatinine are decreased, in some patients, AKI is not captured. Our study also noted that patients with malnutrition have 43% higher odds of developing sepsis. Patients with malnutrition have lower immunity secondary to inadequate dietary intake and high energy requirements, as suggested by Katona et al. [33]. It has also been documented that patients with malnutrition who develop sepsis are at higher odds of developing in-hospital mortality [34]. Our study notes that patients with malnutrition are at higher risk of worse outcomes, and it underscores the need for appropriate nutrition in these patients.

Patients with malnutrition were also noted to have a prolonged LOS and higher hospitalization resource utilization. This is likely due to increased severity, as evidenced by higher rates of death, AKI, ICU admissions, and DVT. Management of these patients with severe disease might require additional management and coordination of care. It is likely that patients with malnutrition are at increased risk of being referred to nursing care facilities. Bell et al. also reported that more than 20% of nursing home residents had malnutrition. The discharge planning for these patients may require coordination between physicians, social workers, and nursing homes, which may further prolong their length of stay [34].

Our study has the following limitations: The NIS database does not contain information regarding the severity of the disease or the methods to establish a diagnosis. It lacks information on the pharmaceutical therapies used and the dietary compliance of these patients. Since NIS uses ICD-10 codes to identify patients, coding errors cannot be excluded. Finally, this database only includes present hospitalization data, and therefore, readmissions cannot be evaluated. Our study’s strengths of large population size and the exclusion of regional bias outweigh the limitations. The findings of this study should be validated in a prospective cohort study that captures more detailed clinical information about compliance, treatment, and long-term mortality.

## Conclusions

Our study reveals that the prevalence of malnutrition in patients with CD is 8.3% among all hospitalized patients. There is a positive association between malnutrition and worse outcomes such as all-cause mortality, sepsis, DVT, and resource utilization. Physicians should be aware of this association, and appropriate screening should be performed on patients with malnutrition. A multidisciplinary approach encompassing nutritional screening, dietary recommendations, medical therapy, and options such as enteral or parenteral nutrition might be beneficial in improving outcomes in these at-risk populations. 
